# Long non-coding RNA HOXA-AS3 promotes cell proliferation of oral squamous cell carcinoma through sponging microRNA miR-218-5p

**DOI:** 10.1080/21655979.2021.1978196

**Published:** 2021-10-26

**Authors:** Yue Zhao, Rui Yao

**Affiliations:** Department of Pediatric Stomatology, Tianjin Stomatological Hospital, Tianjin, China

**Keywords:** Oral squamous cell carcinoma, LncRNA, HOXA-AS3, miR-218-5p, proliferation

## Abstract

Increasing evidence demonstrated long non-coding RNAs (lncRNAs) play important roles in the occurrence and development of oral squamous cell carcinoma (OSCC). This study aimed to explore the role and molecular mechanism of lncRNA HOXA-AS3 in the progression of OSCC. Here, we found that **t**he expression of lncRNA HOXA-AS3 was upregulated in OSCC tissues and cell lines compared with the para-cancerous tissues and normal human oral keratinocyte (NHOK), respectively. Inhibition of HOXA-AS3 significantly inhibited the proliferation and colony formation of OSCC cells. Further, the luciferase reporter assay showed that HOXA-AS3 was directly bound to miR-218-5p. Moreover, the expression of miR-218-5p was negatively regulated by HOXA-AS3, and miR-218-5p could inhibit the expression of collagen type I alpha1 (COL1A1) and lysophosphatidylcholine acyltransferase 1 (LPCAT1). In addition, silencing miR-218-5p reversed the inhibitory effect of HOXA-AS3 knockdown on the proliferative potential of OSCC cells. In summary, our study illustrated that HOXA-AS3 promoted cancer cell proliferation in OSCC, possibly by sponging miR-218-5p for the first time, which provides a new target or a potential diagnostic biomarker for OSCC.

## Introduction

Oral squamous cell carcinoma (OSCC) is one of the most common head and neck squamous cell carcinomas and a public health threat. There are about 500,000 new cases in the world every year, and the incidence is increasing year by year [1,2]. Due to quick progression, invasive growth, easy lymph node, and distance metastasis, and high recurrence rate, the prognosis of OSCC is relatively poor, with an overall 5-year survival rate of less than 50% [3]. In recent years, despite synthetic serial treatments including surgery, radiotherapy, and chemotherapy have been widely applied in patients with OSCC, the cure rate is still less than 50% [4]. Therefore, an improved understanding of the molecular mechanisms underlying OSCC tumorigenesis is necessary to provide novel insights into the pathogenesis of OSCC and thus improve the diagnostic and therapeutic methods.

LncRNAs are a family of RNAs with more than 200 nucleotides in length which do not code for proteins [5]. A growing number of studies have recently demonstrated that aberrant expression of lncRNAs play vital roles in many different types of human cancer, including OSCC, osteosarcoma, hepatocellular carcinoma, nasopharyngeal carcinoma, and gastric cancer [6]. LncRNAs are associated with the biological characteristics of malignant tumors, such as oncogenesis, development, and metastasis [7,8]. Increasing evidence has demonstrated the important roles of lncRNAs in regulating the biological performances of OSCC [9]. For example, the expression of lncRNA HOTAIR influenced the proliferation, apoptosis, and cell cycle in OSCC cells [10]. LncRNA AC132217.4 was significantly upregulated and promoted the cell migration and epithelial-mesenchymal transition through the insulin like growth factor 2 (IGF2) signaling pathway in OSCC [11]. Upregulated lncRNA NEAT1 was correlated with the advanced TNM stage and poor survival of patients and promoted the proliferation and invasion of OSCC cells *in vitro and in vivo* through the NEAT1/miR-365/RGS20 axis [12]. LncRNA LACAT1 was markedly increased in OSCC tissues and cells and promoted the malignant progression of OSCC by regulating miR-4301 [13].

Previous studies have demonstrated that lncRNA HOXA-AS3 is an important gene in tumorigenesis and tumor progression [14–17]. However, its role in OSCC remains unknown. In the present study, we aimed to investigate the expression pattern and role of HOXA-AS3 and further reveal its regulatory mechanism in promoting OSCC progression.

## Materials and methods

### Patient tissue samples

In this study, a total of 52 patients with OSCC who underwent surgical treatment in Tianjin Stomatological Hospital were enrolled, 38 of whom had paired carcinomas and para-cancer tissues, while 14 had only carcinomas tissues. All histopathological types were confirmed by two pathologists. Paracancer tissue refers to normal oral mucosal tissue that was more than 2 cm away from the cancer tissue and does not contain obvious cancer cells. The patients were referred to the 8th edition of UICC/AJCC oral squamous cell carcinoma tumor node metastasis (TNM) staging criteria. Moreover, none of the patients received radiotherapy or chemotherapy before the operation. Clinical characteristics and demographics of the patients in this study were summarized in (Table 1). All patients signed informed consent before using the tissues for this study according to the principles of the Declaration of Helsinki. The tumor tissues were immediately frozen in liquid nitrogen and then stored at −80°C for further research. This study was approved by the Ethics Committee of Tianjin Stomatological Hospital (Tianjin, China).

**Table 1.** Clinical characteristics of patients with OSCC and correlations with HOXA-AS3 expression. (*n* = 52)

Variable	Total	HOXA-AS3 expression		P-value
		Low	High	
Age (years), n (%)				
<60	22 (42.3)	12	10	0.395
≥60	30 (57.7)	12	18	
Gender, n (%)				0.573
Male	32 (61.5)	15	17	
Female	20 (39.5)	9	11	
Smoking history, n (%)				0.711
Yes	28 (53.8)	12	16	
No	24 (46.2)	12	12	
Differentiation, n (%)				0.003*
Poorly differentiated	31 (59.6)	9	22	
Well/moderately differentiated	21 (40.4)	15	6	
TNM stage, n (%)				0.011*
I + II	30 (57.7)	19	11	
III + IV	22 (42.3)	5	17	
Lymph node metastasis, n (%)				0.098
Yes	17 (32.7)	5	12	
No	35 (67.3)	19	16	

Smoking: More than one cigarette a day, sustaining more than 6 months

Drinking: At least once a week, sustaining more than 6 months

* Significant difference (*P* < 0.05).

### Cell culture and transfection

Four OSCC cell lines (TSCCA, CAL-27, SCC-9, and Tca8113) and NHOK cells were purchased from the Cell Bank of Type Culture Collection of the Chinese Academy of Sciences (Shanghai, PRC). All cells were cultured in Dulbecco’s modified Eagle’s medium (DMEM) supplemented with 10% fetal bovine serum (FBS; Gibco, Waltham, MA, USA), 100 IU/mL of penicillin, and 100 μg/mL of streptomycin at 37°C incubator with 5% CO_2_.

shRNA containing the HOXA-AS3 interference sequence (sh-HOXA-AS3) and negative control (sh-NC) were purchased from GeneChem (Shanghai, PRC) and miR-218-5p mimics, anti-miR-218-5p and negative control (miR-NC) were purchased from RiboBio (Guangzhou, PRC). Transfection was performed using Lipofectamine® 2000 Reagent (Invitrogen, Waltham, MA, USA) according to the manufacturer’s protocol in OSCC cells. The culture medium was replaced 6 h after transfection, and transfection efficiency was examined with the expression vector of red fluorescent protein (RFP) at 48 h after the transfection.

### RNA extraction and quantitative real time-polymerase chain reaction (qRT-PCR)

Total RNA was extracted from OSCC tissues or cells using the TRIzol Reagent (Invitrogen, Carlsbad, CA, USA) according to the manufacturer’s instructions. qRT-PCR was performed using the All-in-One™ miRNA qRT-PCR detection kit (GeneCopoeia, Rockville, MD, USA) for miR-218-5p and U6 as the internal control. The relative expression level of mRNA was detected using SYBR Green qRT-PCR assay (Bio-Rad Laboratories Inc, Hercules, CA, USA), and GAPDH was used as the internal control. All qRT-PCR procedure was performed on the ABI 7500 thermocycler (Thermo Fisher Scientific, Waltham, MA, USA). The sequences of the primers were listed in (Table 2). The specific primers for miR-218-5p and U6 were purchased from RiboBio (Guangzhou, PRC). The relative expression levels of detective genes were calculated using the 2^−ΔΔCt^ method.

**Table 2.** The primers sequences used in the study

Gene	Sequence
HOXA-AS3	F: 5ʹ-GCTGAATTAACGGTGGCTCC-3ʹ R:5ʹ-ATGGCGAGCGAAGGGAAG-3’
GAPDH	F: 5ʹ-GGAATCCACTGGCGTCTTCA-3ʹ R: 5ʹ-GGTTCACGCCCATCACAAAC-3’
miR-218-5p	F: 5ʹ- AAGACACCCTGGACGAAGCC −3ʹ R: 5ʹ- ACAACCAGAGTCCACCGGCG −3ʹ
U6	F: 5ʹ-GCTCGCTTCGGCAGCACA-3ʹ R: 5ʹAACGCTTCACGAATTTGCGTG-3ʹ

### Cell proliferation analysis

CCK-8 reagent (Dojindo, Kumamoto, Japan) was used to measure the proliferation of OSCC cells. The OSCC cells at a density of 1 × 10^3^ cells/well were seeded into 96-well plates after transfection for 48 h. After culturing for 12 h, 24 h, 48 h, and 72 h respectively, and CCK-8 reagent was added to each well with 10 μl and further incubated for 4 h. The optical density (OD) value of each well was detected using an enzyme labeling instrument at 450 nm.

### Colony formation assay

Colony formation assay was performed as previously reported [18]. The OSCC cells were inoculated into 6-well plates with 200 cells per well 48 h after transfection for 48 h. Subsequently, the cells were cultured in the complete medium for 14 d. The medium was replaced after 5 d and then replaced every 3 d. When the cell colonies formed, the medium was sucked dry. Then, the cells were washed twice with phosphate-buffered saline (PBS) and fixed with 4% paraformaldehyde at 4°C for 1 h. Next, the cells were stained with 0.1% crystal violet staining solution for 20 min. Finally, the number of cell colonies containing >50 cells in each well was calculated and photographed.

### Luciferase reporter assay

Online Software Starbase v2.0 (http://starbase.sysu.edu.cn) was used to predict the target miRNAs of lncRNA HOXA-AS3. The wild-type HOXA-AS3 containing the miR-218-5p seed sequence fragment (HOXA-AS3 Wt) and mutant-type (HOXA-AS3 Mut) luciferase vectors were conducted. The luciferase reporter assay was conducted as previously reported [7]. The OSCC cells (5 × 10^3^ cells) were seeded into 48-well plates and were co-transfected with the luciferase vectors and miR-218-5p mimics or negative control using Lipofectamine® 2000 Reagent for 48 h. This assay was normalized with 0.05 μg of the RFP expression vector pDsRed2-N1 (Clontech, USA). Subsequently, cells were lysed with RIPA lysis buffer, and the luciferase activity and RFP intensity were detected with the F-4500 Fluorescence Spectrophotometer (Hitachi, Japan) according to the manufacturer’s instructions.

### RNA immunoprecipitation (RIP) assay

The RIP assay was performed as reported by Zhang Y et al. [19]. RIP assay was used to detect the sponge function of HOXA-AS3 on miR-218-5p by using Magna RIPTM RNA Immunoprecipitation Kit (Millipore, Bedford, MA, USA). Briefly, the OSCC cells (5 × 10^3^ cells) were seeded into 48-well plates and transfected with miR-218-5p mimics, Vector-HOXA-AS3, or corresponding controls for 48 h, and then were lysed using the lysis buffer. Next, cell lysates were incubated with anti-Ago2 (Abcam, UK) or anti-IgG (Abcam, UK) and protein A/G magnetic beads. Finally, co-precipitated RNAs were detected by qRT-PCR.

### Statistical analysis

Statistical analysis was performed using SPSS version 20.0 (IBM, Armonk, NY, USA). Measurement data conforming to normal distribution are presented as the mean ± standard deviation. Differences among multiple groups were analyzed by ANOVA (one-way) followed by Tukey *t*-test, and differences between the two groups were analyzed using the student’s *t*-test. Correlation analysis between HOXA-AS3 and miR-218-5p expression was assessed using Pearson’s correlation coefficient. The prognosis survival time of patients was evaluated using Kaplan-Meier analysis, and the Log-rank test was used to examine the difference between different curves. Each experiment was repeated three times independently. *P* < 0.05 was considered to indicate a statistically significant difference.

## Results

Here, we aimed to investigate the role and molecular mechanism of lncRNA HOXA-AS3 in OSCC progression. First, we examined the expression levels of HOXA-AS3 in OSCC tissues and cells. Then, the effect of knocking down HOXA-AS3 on cell proliferative activity was evaluated. Further, bioinformatics and cell experiments were used to reveal the mechanism of HOXA-AS3.

### LncRNA HOXA-AS3 was upregulated in OSCC tissues and associated with a poor prognosis

**Figure 1.**
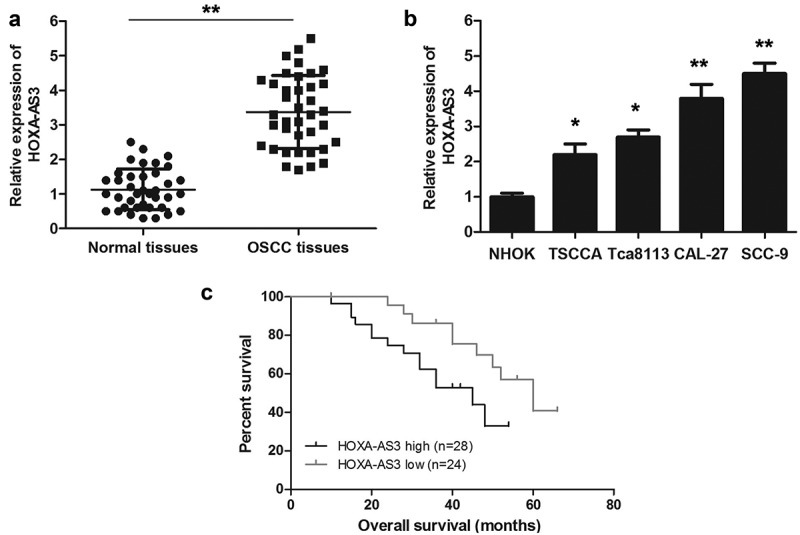
HOXA-AS3 expression is upregulated in OSCC tissues and cell lines. (a) The expression of HOXA-AS3 in OSCC tissues and para-cancerous tissues was measured using qRT-PCR. (b) Expression levels of HOXA-AS3 in NHOK and OSCC cell lines (TSCCA, CAL-27, SCC-9, and Tca8113) were detected via qRT-PCR. (c) The Kaplan-Meier survival curve indicated that the prognosis of patients in HOXA-AS3 high-expression group was significantly worse than that of patients in HOXA-AS3 low-expression group. *P < 0.05, **P < 0.01

In this study, we examined the expression of lncRNA HOXA-AS3 in 38 paired human OSCC tissues and the corresponding para-carcinoma tissues and OSCC cell lines using qRT-PCR assay. As shown in (Figure 1(a)), lncRNA HOXA-AS3 was significantly overexpressed in OSCC tissues compared with para-carcinoma tissues. Meanwhile, the expression of lncRNA HOXA-AS3 in four OSCC cell lines was obviously higher than that in NHOK cells (Figure 1(b)). Further, we divided 52 OSCC patients into HOXA-AS3 high expression group and HOXA-AS3 low expression group according to the mean relative level of lncRNA HOXA-AS3 expression. The associations between lncRNA HOXA-AS3 expression and the clinicopathological characteristics of OSCC patients were summarized in (Table 1). LncRNA HOXA-AS3 was overexpressed in OSCC with poor differentiation (*P* < 0.01) and a high TNM stage (*P* < 0.05). Moreover, as shown in (Figure 1(c)), the lncRNA HOXA-AS3 expression in the 38 OSCC tissues was significantly associated with the overall survival of OSCC patients (*P* < 0.05). The patients with lower HOXA-AS3 expression had longer overall survival compared to those with higher HOXA-AS3 expression.

### Knockdown of lncRNA HOXA-AS3 inhibited the growth of OSCC cells

**Figure 2.**
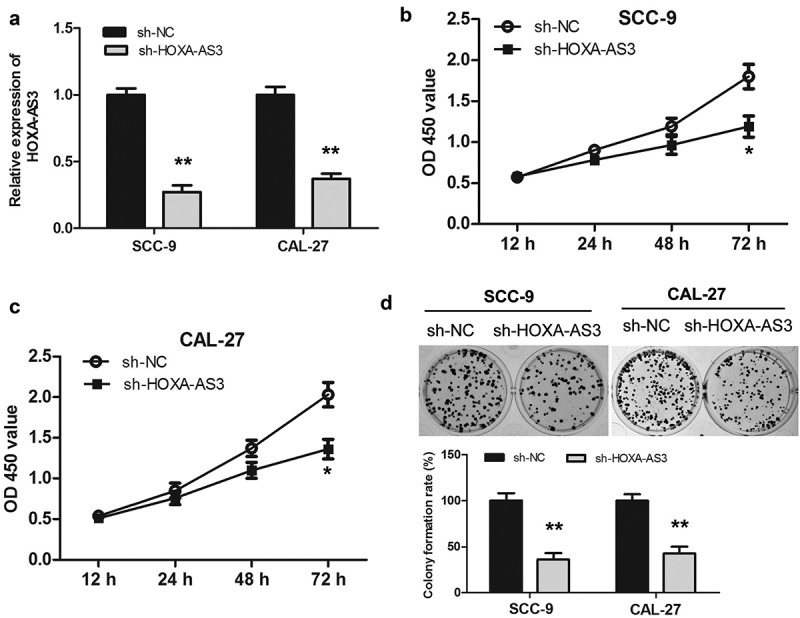
HOXA-AS3 knockdown inhibited OSCC cell proliferation and colony formation in vitro and in vivo. (a) Transfection efficacy of sh-HOXA-AS3 in SCC-9 and CAL-27 cells. (b, c) Cell Counting Kit-8 assay showed that HOXA-AS3 knockdown inhibited cell proliferation in SCC-9 and CAL-27 cells. (d) Colony formation assay showed that HOXA-AS3 knockdown significantly reduced the number of colonies. *P < 0.05, **P < 0.01

Among the four selected OSCC cell lines, SCC-9 and CAL-27 cells expressed the relatively high level of lncRNA HOXA-AS3 (Figure 1(b)), which were selected for transfection and subsequent experiments. Three shRNAs directed against HOXA-AS3 were designed and synthesized in our experiment. These shRNAs were transfected into SCC-9 and CAL-27 cells and changes in lncRNAs HOXA-AS3 expression were evaluated. According to the qRT-PCR results, The shRNA targeting HOXA-AS3 with the best inhibitory effect was selected for subsequent experiments (Supplementary Figure S1). As shown in (Figure 2(a)), sh-HOXA-AS3 significantly decreased the HOXA-AS3 expression compared with the sh-NC group in the SCC-9 and CAL-27 cells. CCK-8 data revealed that downregulation of HOXA-AS3 observably inhibited the proliferation of SCC-9 and CAL-27 cells compared with the sh-NC group (Figure 2(b,c)). Furthermore, we found that the downregulation of HOXA-AS3 decreased the colony formation ability of SCC-9 and CAL-27 cells by the colony formation assay (Figure 2(d)). Thus, it was concluded that the downregulation of lncRNA HOXA-AS3 suppressed the cell growth of OSCC.

### LncRNA HOXA-AS3 could directly bind miR-218-5p in OSCC

**Figure 3.**
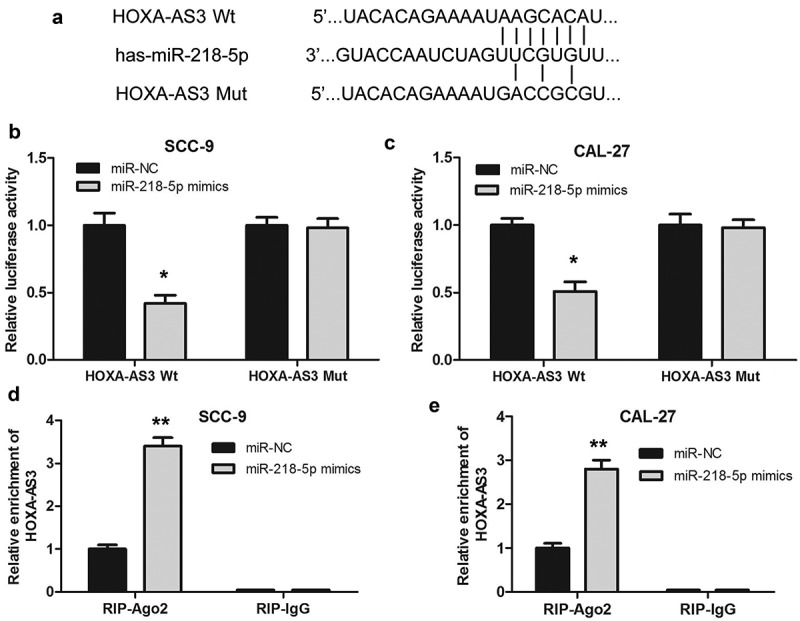
HOXA-AS3 directly interacted with miR-218-5p. (a) Predicted binding of human miR-218-5p with the wild-type 3ʹUTR region of HOXA-AS3 mRNA and a mutated 3ʹUTR of HOXA-AS3. (b, c) Luciferase reporter gene assay verified that HOXA-AS3 could directly bind to miR-218-5p in SCC-9 and CAL-27 cells. (d, e) SCC-9 and CAL-27 cells were transfected with miR-218-5p mimics or control, followed by the measurement of HOXA-AS3 mRNA enrichment with anti-Ago2 by qRT-PCR, and anti-IgG used as control. *P < 0.05, **P < 0.01

To explore the possible mechanism of lncRNA HOXA-AS3 in regulating the biological behaviors of OSCC cells, we predicted the binding sites of lncRNA HOXA-AS3 using Online Software Starbase v2.0 [20]. As shown in (Figure 3(a)), lncRNA HOXA-AS3 could bind to miR-218-5p. Subsequently, we validated the targeted effect of miR-218-5p on HOXA-AS3 via the luciferase reporter assay. The results revealed that luciferase activity significantly decreased in OSCC cells co-transfected with miR-218-5p mimics and HOXA-AS3 Wt; however, there has no marked change of luciferase activity in the mutated HOXA-AS3 group, demonstrating the binding between miR-218-5p and HOXA-AS3 (Figure 3(b,c)). Furthermore, the RIP assay revealed that HOXA-AS3 was substantially enriched by miR-218-5p overexpression with anti-Ago2 in OSCC cells. The data suggested an endogenous interaction between HOXA-AS3 and miR-218-5p, indicating that HOXA-AS3 might work as a miR-218-5p sponge (Figure 3(d,e)). In short, these results suggested that lncRNA HOXA-AS3 could directly bind miR-218-5p in OSCC.

### miR-218-5p was downregulated in human OSCC and inversely correlated with HOXA-AS3 expression

**Figure 4.**
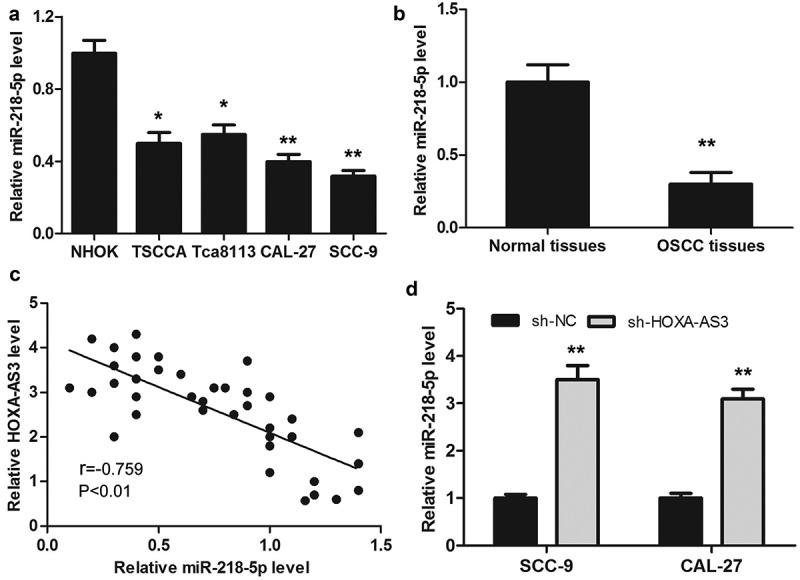
MiR-218-5p was downregulated in OSCC tissues and cells and inversely correlated with HOXA-AS3 expression. (a) The expression of miR-218-5p in OSCC cell lines and NHOK was detected by qRT-PCR. (b) The expression of miR-218-5p in OSCC tissues and para-cancerous tissues was detected by qRT-PCR. (c) HOXA-AS3 and miR-218-5p expression level was negatively correlated in OSCC tissues (r = −0.759, *P* < 0.01, n = 38). (d) qRT-PCR was used to measure the expression level of miR-218-5p after HOXA-AS3 knockdown in OSCC cell lines. *P < 0.05, **P < 0.01

QRT-PCR was performed to detect the expression level of miR-218-5p in OSCC cell lines and 38 pairs of OSCC and para-cancerous tissues. As shown in (Figure 4(a)), the expression level of miR-218-5p was markedly lower in OSCC cells than that of NHOK cells. Meanwhile, the expression of miR-218-5p was remarkably lower in OSCC tissues compared with that of para-cancerous tissues (Figure 4(b)). And then, Pearson’s correlation analysis showed a correlation between HOXA-AS3 and miR-218-5p expression in OSCC tissues. As shown in (Figure 4(c)), the expression of miR-218-5p was inversely correlated with HOXA-AS3 expression level in OSCC tissues. Furthermore, the expression of miR-218-5p was markedly increased after HOXA-AS3 knockdown (Figure 4(d)).

### Overexpression of miR-218-5p inhibited the proliferation of OSCC cells

**Figure 5.**
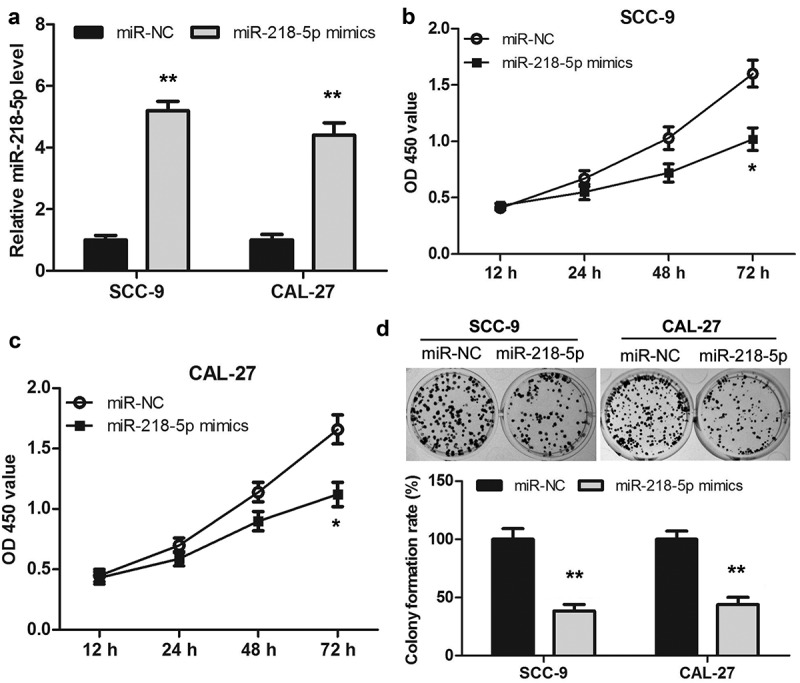
Overexpression of miR-218-5p inhibited the proliferation of OSCC cells. (a) Transfection efficacy of miR-218-5p mimics in SCC-9 and CAL-27 cells. (b, c) Cell Counting Kit-8 assay showed that overexpression of miR-218-5p inhibited cell proliferation in OSCC cells. (d) Colony formation assay showed that overexpression of miR-218-5p significantly reduced the number of colonies. *P < 0.05, **P < 0.01

To investigate the role of miR-218-5p in the proliferation of OSCC cells, miR-218-5p mimics or miR-NC was transfected into SCC-9 and CAL-27 cells. As shown in (Figure 5(a)), the expression level of miR-218-5p was significantly increased in the miR-218-5p mimics group compared to that of the miR-NC group both in SCC-9 and CAL-27 cells. Then, the proliferation of OSCC cells was measured using CCK-8 and colony formation assay, respectively. The data revealed that the proliferation ability of SCC-9 and CAL-27 cells in the miR-218-5p mimics group was reduced when compared to the miR-NC group (Figure 5(b-d)). Together, overexpression of miR-218-5p inhibited the OSCC cell proliferation, which was consistent with the downregulation of HOXA-AS3.

### miR-218-5p regulates the expression of COL1A1 and LPCAT1 in OSCC cells

**Figure 6.**
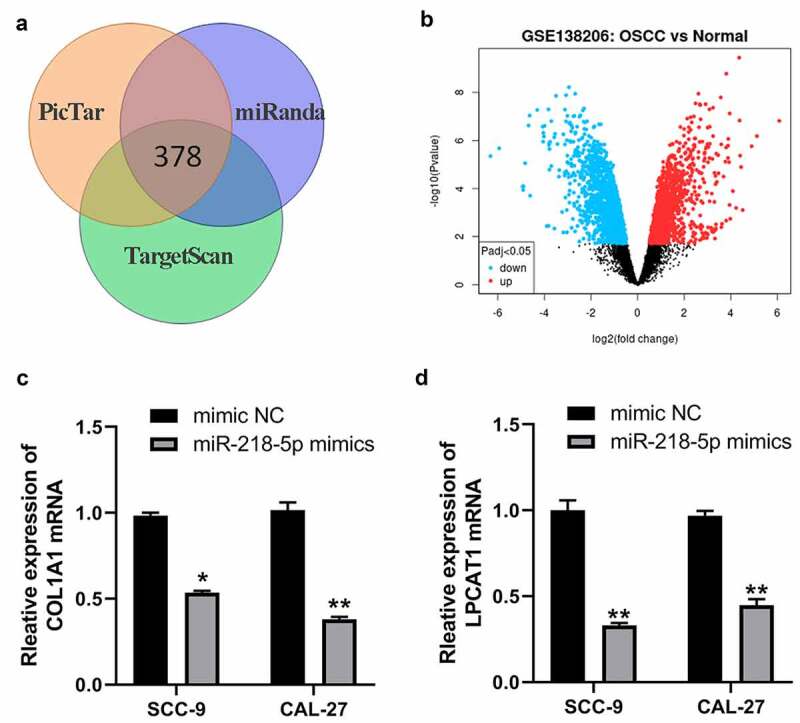
MiR-218-5p inhibited COL1A1 and LPCAT1 in OSCC cells. (a) The poteintial targeted mRNAs of miR-218-5p were predicted by PicTar, miRanda and TargetScan online databases. (b) Volcano plot showing the DEGs identified from GSE138206. X axis represents log transformed P value, and Y axis indicates the mean expression differences of genes between OSCC samples and normal samples. (c) qRT-PCR was used to measure the expression of COL1A1 after miR-218-5p overexpression in OSCC-9 cells. (d) qRT-PCR was used to measure the expression of LPCAT1 after miR-218-5p overexpression in OSCC cells. *P < 0.05, **P < 0.01, ***P < 0.001

Previous studies have demonstrated miRNAs exert their functions mainly by binding to the mRNAs 3ʹUTR region to inhibit mRNAs levels. By PicTar, miRanda and TargetScan online databases, we obtained 378 potential targeted genes of miR-218-5p (Figure 6(a)). Meantime, we analyzed GSE138206, a gene expression microarray regarding OSCC in the GEO database and screened differentially expressed genes (DEGs) by using GEO2R (|log_2_FC| > 1.5 and adj. p-value < 0.05). These DEGs from GSE138206 datasets were shown in Figure 6(b) and 275 up-regulated DEGs in OSCC were obtained. Then, 378 potential target genes of miR-218-5p were intersected with 275 up-regulated DEGs in OSCC, and FLRT3, TNC, COL1A1, LPCAT1 and HOXA1 were found to be potential target genes of miR-218-5p in OSCC. Previous studies have demonstrated that COL1A1 and LPCAT1 were overexpressed in OSCC and their overexpression promote proliferation and invasion of OSCC cells [21,22]. Therefore, we speculated whether COL1A1 and LPCAT1 were regulated by miR-218-5p. The qRT-PCR results showed that miR-218-5p mimics decreased the expression of COL1A1 and LPCAT1 mRNA in SCC-9 and CAL-27 cells (Figure 6(c,d)). Moreover, knockdown of HOXA-AS3 also led to decreased levels of COL1A1 and LPCAT1 mRNA in OSCC cells (Figure 6(e,f)).

### Inhibition of miR-218-5p reversed the effect of lncRNA HOXA-AS3 knockdown on the proliferation of OSCC cells

**Figure 7.**
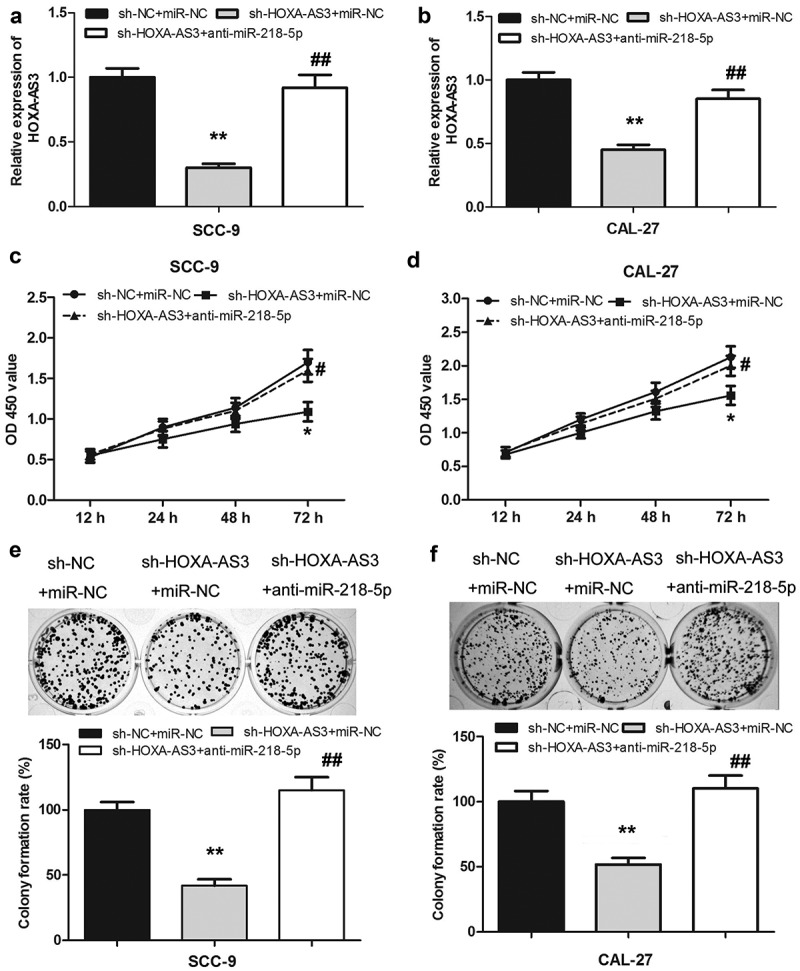
LncRNA HOXA-AS3 promoted OSCC development through regulating miR-218-5p. (a, b) The expression level of HOXA-AS3 in cells co-transfected with sh-HOXA-AS3 and anti-miR-218-5p was detected by qRT-PCR. (c-f) The inhibited proliferation of SCC-9 and CAL-27 cells by HOXA-AS3 knockdown was reversed by anti-miR-218-5p. *P < 0.05, **P < 0.01

To further study the interaction between HOXA-AS3 and miR-218-5p in OSCC cells, anti-miR-218-5p was transfected into SCC-9 and CAL-27 cells with HOXA-AS3 silencing. The expression level of HOXA-AS3 in each group was measured by qRT-PCR. As shown in (Figure 7(a,b)), the expression of HOXA-AS3 in the co-transfected with si-HOXA-AS3 and anti-miR-218-5p group was observably higher than that of the co-transfected with si-HOXA-AS3 and miR-NC group. Moreover, the CCK-8 assay showed that HOXA-AS3 knockdown could significantly suppress the proliferative activity of SCC-9 and CAL-27 cells but was further reversed by miR-218-5p knockdown (Figure 7(c,d)). As shown in (Figure 6(e,f)), colony formation assay yielded identical results.

## Discussion

OSCC is a kind of malignant tumor that seriously threatens human health, and it has the characteristics of easy invasion and lymph node metastasis. Although great progress has been made in exploring new treatments, the prognosis of OSCC is still unsatisfactory due to its high malignancy [23]. Risk factors for OSCC include betel nut, tobacco, low-quality edible pigment, human papillomavirus infection, etc [24,25]. At present, the pathogenesis of OSCC is still not very clear, which may involve a multi-gene epigenetic and metabolic process, such as the loss of the function of cancer suppressor genes and activation of function of oncogenes [26]. Thus, the research on the molecular biological mechanism of OSCC, especially the detection and diagnosis of specific early tumor markers, is important for the early diagnosis and prognosis of OSCC patients.

HOXA-AS3 is a novel lncRNA located in chromosome 7p15.2 and belongs to the clusters of HOX genes, a group of highly homologous transcription factors that regulate embryological development [27]. HOXA-AS3 interacts with Enhancer Of Zeste 2 (EZH2) and acts as an epigenetic switch that determines the lineage specification of mesenchymal stem cells [28]. Previous studies reports that upregulation of lncRNA HOXA-AS3 was observed in several cancers and promotes tumor progression and predicts poor prognosis [14,16,29–31]. For example, Wu et al. [32] reported that the expression of HOXA-AS3 was significantly increased in glioma tissues and cell lines, and knockdown of HOXA-AS3 inhibited the cell growth in vitro and vivo, promoted cell apoptosis, and impaired cell migration in glioma cells. Similarly, HOXA-AS3 was markedly upregulated in lung adenocarcinoma tissues and cells and promoted cancer cell progression [33]. However, there is no report on the function and molecular mechanism of HOXA-AS3 in OSCC. In this study, the expression levels of lncRNA HOXA-AS3 in OSCC tissues and cell lines were measured. We discovered that lncRNA HOXA-AS3 was more highly expressed in OSCC tissues and cells than in para-carcinoma tissues and NHOK cells. Moreover, the high expression of HOXA-AS3 was obviously correlated with the pathological stage and overall survival of OSCC patients. To further investigate the function of lncRNA HOXA-AS3 on the biological performances of OSCC cells, the HOXA-AS3 knockdown model was constructed in the SCC-9 and CAL-27 cells. The data revealed that knockdown of HOXA-AS3 suppressed the cell proliferation and growth in OSCC. This means that the high expression of HOXA-AS3 might be closely related to the progression of OSCC.

Currently, lncRNAs have been demonstrated to function as competing endogenous RNAs (ceRNA) by sponging miRNA and inhibiting intracellular miRNA function [31]. Therefore, establishing the interrelationship of lncRNA and its regulation of miRNA may help further understand the molecular mechanism underlying tumor progression and provide potential therapeutic targets for the clinical treatment of tumors. Bioinformatics analysis predicted a potential binding site of miR-218-5p in HOXA-AS3, and luciferase reporter assay confirmed the direct interaction in the SCC-9 and CAL-27 cells. Meanwhile, the expression of miR-218-5p was markedly decreased in OSCC tissues compared with para-carcinoma tissues and was negatively correlated with HOXA-AS3 expression. RIP assay showed that HOXA-AS3 might work as a miR-218-5p sponge in OSCC. These results suggested that lncRNA HOXA-AS3 might influence the function of OSCC cells *via* sponging and regulating the miR-218-5p.

Previous studies have demonstrated that miR-218-5p is a tumor suppressor miRNA in various types of cancers [34–37]. Li X et al. [38] showed that miR-218-5p was downregulated in invasion front cells and negatively regulates OSCC invasiveness by targeting the CD44-ROCK pathway. Our study showed that the expression of miR-218-5p was significantly downregulated in OSCC cell lines, and overexpression of miR-218-5p remarkably inhibited the proliferation and colony formation of OSCC cells. Further, using bioinformatics techniques, we found that COL1A1 and LPCAT1 may be potential target genes of miR-218-5p in OSCC. COL1A1 is the major component of type I collagen. aberrant expression of COL1A1 is implicated in numerous cancers. He B et al. [39]showed that COL1A1 was low-expressed in OSCC and the down-regulation of COL1A1 suppressed the proliferation, invasion, and mitosis of OSCC cells. LPCAT1 is a cytoplasmic enzyme that catalyzes the conversion of lysophosphatidylcholine (LPC) to phosphatidylcholine (PC), thereby remodeling the PC biosynthetic pathway. So far, LPCAT1 overexpression has been reported to promote cancer progression, metastasis and recurrence in multiple tumors. Spenlé C et al. [40] reported that LPCAT1 mRNA and protein were up-regulated in OSCC and a strong correlation between LPCAT1-positive OSCCs and tumoral size and regional lymph node metastasis. LPCAT1 knockdown decreased significantly cellular proliferation and invasiveness, cellular migration. In this study, COL1A1 and LPCAT1 were positively regulated by HOXA-AS3 and negatively regulated by miR-218-5p mimics. These data indicate that the role of HOXA-AS3/miR-218-5p in OSCC may be mediated by regulating the expression of COL1A1 and LPCAT1.

To investigate whether HOXA-AS3 promoted the development of OSCC through regulating miR-218-5p, anti-miR-218-5p was transfected into SCC-9 and CAL-27 cells with knockdown of HOXA-AS3. The results revealed that knockdown of miR-218-5p could restore OSCC cell proliferation and colony formation activities after HOXA-AS3 silencing, suggesting that HOXA-AS3 might promote malignant progression of OSCC by inhibiting miR-218-5p.

## Conclusion

In summary, our study shows that lncRNA HOXA-AS3 is significantly upregulated in OSCC and this high expression positively correlated with the pathological stage and poor prognosis of patients, which promotes the development of OSCC through sponging and inhibiting miR-218-5p.

## Supplementary Material

Supplemental Material

## Data Availability

The datasets during and analyzed during the current study are available from the corresponding author on reasonable request.
