# Supplementary figures and images for: Long non-coding RNA HOXA-AS3 promotes cell proliferation of oral squamous cell carcinoma through sponging microRNA miR-218-5p
Source: Bioengineered. 2021 Oct 26;12(1):8724–37. doi: 10.1080/21655979.2021.1978196 (PMC8806885; doi:10.1080/21655979.2021.1978196)

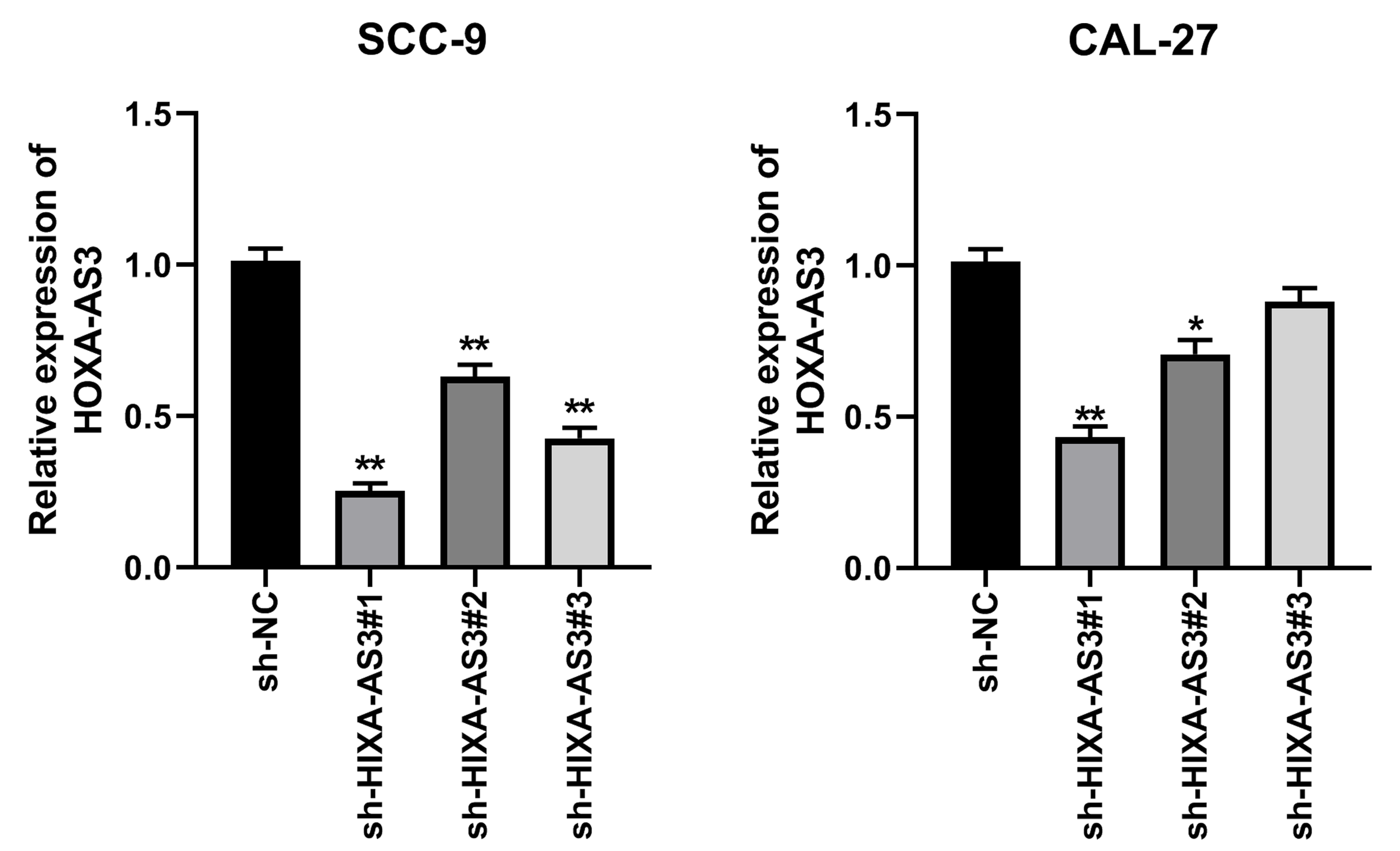

Supplement: Supplemental Material [file KBIE_A_1978196_SM3647.tif]
